# Use of the growing environment as a source of variation to identify the quantitative trait transcripts and modules of co-expressed genes that determine chlorogenic acid accumulation

**DOI:** 10.1111/j.1365-3040.2010.02141.x

**Published:** 2010-07

**Authors:** THIERRY JOËT, JORDI SALMONA, ANDRÉINA LAFFARGUE, FRÉDÉRIC DESCROIX, STÉPHANE DUSSERT

**Affiliations:** 1 IRD, UMR DIAPC, Pôle de Protection des Plantes97410 Saint Pierre, La Réunion, France; 2 IRD, UMR DIAPCBP 64501, 34394 Montpellier, France; 3 CIRAD, UMR QualiSud97410, Saint Pierre, La Réunion, France

**Keywords:** *Coffea*, albuminous seed, caffeoyl quinic acid, co-expression network, endosperm, feruloyl quinic acid, phenylpropanoid, temperature, transcriptome

## Abstract

Developing *Coffea arabica* seeds accumulate large amounts of chlorogenic acids (CGAs) as a storage form of phenylpropanoid derivatives, making coffee a valuable model to investigate the metabolism of these widespread plant phenolics. However, developmental and environmental regulations of CGA metabolism are poorly understood. In the present work, the expression of selected phenylpropanoid genes, together with CGA isomer profiles, was monitored throughout seed development across a wide set of contrasted natural environments. Although CGA metabolism was controlled by major developmental factors, the mean temperature during seed development had a direct impact on the time-window of CGA biosynthesis, as well as on final CGA isomer composition through subtle transcriptional regulations. We provide evidence that the variability induced by the environment is a useful tool to test whether CGA accumulation is quantitatively modulated at the transcriptional level, hence enabling detection of rate-limiting transcriptional steps [quantitative trait transcripts (QTTs)] for CGA biosynthesis. Variations induced by the environment also enabled a better description of the phenylpropanoid gene transcriptional network throughout seed development, as well as the detection of three temporally distinct modules of quantitatively co-expressed genes. Finally, analysis of metabolite-to-metabolite relationships revealed new biochemical characteristics of the isomerization steps that remain uncharacterized at the gene level.

## INTRODUCTION

Phenylpropanoid-derived compounds are ubiquitous plant secondary metabolites. Among them, esters formed between hydroxycinnamic acids and quinic acid, collectively known as chlorogenic acids (CGAs), which are a major family of soluble plant phenolics (Clifford 1999). They are commonly found in Asteraceae, Solanaceae and Rubiaceae (Molgaard & Ravn 1988). Species of the genus *Coffea*, which belongs to the Rubiaceae family, all accumulate CGAs in their seeds (Campa *et al*. 2005). In *Coffea arabica*, CGAs represent up to 8% of the dry mass (DM) of the mature seed (Farah & Donangelo 2006), which is made up of a copious endosperm (approx. 99% of the seed mass) surrounding a tiny rudimentary embryo. CGAs transiently accumulate to a spectacular extent (15–20% DM) in developing seeds during the early phase of endosperm expansion (Joët *et al*. 2009), meaning the *Coffea* seed is a good model for their biosynthesis and accumulation. CGAs are stored intracellularly, forming vacuolar complexes with caffeine (Môsli-Waldhauser & Baumann 1996). Because the transferase reaction that couples quinic acid to cinnamic acid derivatives is reversible, CGAs are a storage form of cinnamic acid derivatives and are considered as intermediates in the lignin biosynthetic pathway (Aerts & Baumann 1994; Schoch *et al*. 2001). Several other functions have been attributed to CGAs as they exhibit anti-pathogenic, anti-herbivorous and allelopathic properties (Maher *et al*. 1994; Dixon & Paiva 1995). In addition, these compounds have several beneficial health properties largely explained by their antioxidant activity (Grace, Logan & Adams 1998). Finally, in coffee, CGAs also have a major influence on beverage quality and play an important role in the formation of the coffee flavour (for a review, see Farah & Donangelo 2006). The main groups of CGAs found in *C. arabica* seeds include caffeoylquinic acids (CQAs), represented by three main isomers (3-, 4- and 5-CQA), dicaffeoylquinic acids (diCQAs), also with three main isomers (di3,4-CQA, di3,5-CQA and di4,5-CQA) and feruloylquinic acids with two main isomers (4- and 5-FQA). The most abundant CGA is 5-CQA, which represents more than 70% of total CGA.

The first steps of CQA and FQA biosynthesis involve the well-characterized enzymes of the ‘core phenylpropanoid pathway’, namely phenylalanine ammonia-lyase (PAL), cinnamate 4-hydroxylase (C4H) and 4-coumarate CoA ligase (4CL). Then, from *p*-coumaroyl-CoA, two alternative routes have been proposed for the production of 5-CQA (Schoch *et al*. 2001; Niggeweg, Michael & Martin 2004). The first route involves only two enzymatic steps: esterification of quinic acid on *p*-coumaroyl-CoA, then hydroxylation of *p*-coumaroyl quinate to form 5-CQA. The second route requires four steps: esterification of shikimic acid on *p*-coumaroyl-CoA, then hydroxylation of *p*-coumaroyl shikimate to form caffeoyl shikimic acid, followed by its de-esterification to produce caffeoyl-CoA, which in turn is re-esterified with quinic acid to form 5-CQA.

Early experiments using radiolabelled substrates suggested that 5-CQA biosynthesis preferentially uses the first route in coffee and solanaceous species (Steck 1968; Nagels & Parmentier 1976; Colonna 1986). Recently, two paralogous genes, *HCT* and *HQT*, encoding the enzyme that catalyses the esterification/de-esterification steps (hydroxycinnamoyl-CoA shikimate/quinate hydroxycinnamoyl transferase), were evidenced in Solanaceae (Hoffmann *et al*. 2003; Niggeweg, Michael & Martin 2004). Using gene silencing and over-expression, the first route, which requires HQT for esterification, was shown to be the principal route for 5-CQA accumulation in Solanaceae (Niggeweg *et al*. 2004), while the role of HCT could be limited to 5-CQA remobilization, as silenced HCT tobacco plants were shown to accumulate large amounts of 5-CQA in the stem (Hoffmann *et al*. 2004). No gene homologous to *HQT* has been found in *Arabidopsis* (Niggeweg *et al*. 2004). By contrast, both *HCT* and *HQT* genes have been characterized in *Cynara cardunculus*, which belongs to the Asteraceae family (Comino *et al*. 2007, 2009). EST analysis also revealed both *HCT* and *HQT* transcription in developing coffee seeds, suggesting that the sharing of tasks (synthesis/remobilization) between these two transferases might be conserved in CGA accumulating plants. In this respect, it is worth noting that the plant family most closely related to coffee in which extensive sequencing has been conducted is Solanaceae (Lin *et al*. 2005). Finally, recombinant coffee HCT has been shown to be functional in 5-CQA remobilization (Lepelley *et al*. 2007).

Regarding the hydroxylation step, molecular characterization of *p*-coumaroyl ester 3'-hydroxylase (C3'H) was first performed in *Arabidopsis thaliana* (Schoch *et al*. 2001; Franke *et al*. 2002). Even if this model plant does not accumulate CGA, the *in vitro* characterization of this enzyme demonstrated its activity with both *p*-coumaroyl shikimate and *p*-coumaroyl quinate as substrates. In coffee plants, two C3'H genes were recently described, and their recombinant enzymes were functionally characterized. *C3Hc1* was suggested to be involved in CQA biosynthesis, while *C3Hc2* was only active in cinnamic esters of shikimate (Mahesh *et al*. 2007).

The knowledge of genes involved in 5-CQA biosynthesis therefore became sufficient to develop genomic pathway-guided approaches. In coffee, it led to several transcript profiling studies focused on the development of the seed and the pericarp (Koshiro *et al*. 2007; Lepelley *et al*. 2007; Salmona *et al*. 2008; Joët *et al*. 2009). These studies provided a comprehensive timing of 5-CQA accumulation, especially regarding the transition between stages when the perisperm predominated and those when it is replaced by the endosperm (Joët *et al*. 2009). The studies also consistently suggested that 5-CQA biosynthesis might be controlled at the transcriptional level. However, very little is known about the quantitative control of 5-CQA biosynthesis through transcriptional activity. With the exception of the marked increase in *HQT* and *C3'H* mRNA levels reported in UV-C-treated leaves of *C. cardunculus* (Comino *et al*. 2009; Moglia *et al*. 2009), the influence of the environment on 5-CQA biosynthetic gene expression has been poorly studied up to now.

The preferential pathway for FQA biosynthesis in coffee has not been elucidated yet. It could first rely on the feruloyl-CoA pool yielded from caffeoyl-CoA through activity of caffeoyl-CoA 3-*O*-methyl transferase (CCoAOMT; Zhong *et al*. 1998). A feruloyl quinate transferase would then catalyse the *trans*-esterification of feruloyl-CoA with quinate, but it remains to be characterized. It is also still unclear whether the caffeoyl-CoA pool used for feruloyl-CoA synthesis originates directly from the remobilization of 5-CQA through HCT activity, or comes from the shikimate ester route. Alternatively, FQA biosynthesis could rely on direct methylation of 5-CQA by an *O*-methyl transferase enzyme. This one-step FQA biosynthesis pathway has been suggested to occur in coffee leaves (Colonna 1986). In this respect, the recent characterization of a CCoAOMT-like enzyme (AtTSM1) involved in the methylation of phenylpropanoid polyamine conjugates in the inflorescence and tapetum of *Arabidopsis* is worth mentioning (Fellenberg *et al*. 2008; Fellenberg, Böttcher & Vogt 2009). Indeed, the recombinant AtTSM1 enzyme also converts very efficiently 5-CQA into 5-FQA (Fellenberg *et al*. 2008).

The processes involved in 5-CQA and 5-FQA isomerization are also very poorly understood. Di3,5-CQA is formed by caffeate substitution of 5-CQA at the 3-position by a CGA caffeoyl transferase activity. This reaction was first described in *Xanthium* leaves and potato tubers (Taylor & Zucker 1966), and further characterized in sweet potato (Kojima & Kondo 1985; Villegas *et al*. 1987). The isomeric monosubstituted quinic acids, 3-CQA and 4-CQA, 3-FQA and 4-FQA, di3,4-CQA and di4,5-CQA, are thought to be produced by enzymatic *trans*-esterification of 5-CQA, 5FQA and di3,5-CQA, respectively (Hanson 1965). Nothing is known about the enzymes and genes involved in *trans*-esterification of 5-FQA, 5-CQA and di3,5-CQA. Similarly, di3,5-CQA biosynthesis remains to be characterized at the molecular level. A better understanding of these processes through a pathway-guided genomic approach is therefore not possible. Nevertheless, a significant correlation between contents in 5-FQA and 4-FQA isomers was reported in seeds of *Coffea liberica* (Ky *et al*. 1999) and *Coffea pseudozanguebariae* (Bertrand *et al*. 2003), suggesting that identifying genetic or environmental sources of variation might be a reliable preliminary approach to better describe the steps leading to these minor CGAs.

Recently, we demonstrated the dramatic influence of the mean daily temperature on the sugar, lipid and CGA composition of coffee seeds at maturity (Joët *et al*. 2010). These results suggest that temperature is a key environmental parameter that acts directly on CGA metabolism routing. However, how environmental factors and developmental programmes interplay during seed development and maturation remains to be described. In the present work, the expression of selected phenylpropanoid genes, together with the accumulation profiles of CGA isomers, was consequently monitored throughout seed development across 16 locations all over Reunion Island. Furthermore, we assume that the variability induced by the environment is a useful tool to test whether CGA accumulation is quantitatively modulated at the transcriptional level, hence enabling detection of quantitative trait transcripts (QTTs) for the regulation of CGA accumulation. This concept was only recently proposed (Passador-Gurgel *et al*. 2007) and was successfully transposed in plant biology to characterize rate-limiting transcriptional steps in carotenoid biosynthesis (Vallabhaneni & Wurtzel 2009). Moreover, provided that the environment induces variations in transcription level, this design may be a valuable system to identify networks of phenylpropanoid genes that are quantitatively co-expressed or co-repressed throughout development. Many studies combining metabolite and transcript profiling have been carried out to investigate the adaptive response of plants to abiotic stresses, such as low temperature, drought, oxygen deprivation or major nutrient deficiency. By contrast, only a few focused on the primary or secondary metabolism of the developing seed or fruit using field measurements (Blodner *et al*. 2007; Howarth *et al*. 2008; Carbone *et al*. 2009; Wan *et al*. 2009). To our knowledge, the present genomic work is the first to undertake a comprehensive analysis of a plant secondary metabolism across a wide set of contrasted natural environments.

## MATERIALS AND METHODS

### Biological material

Experiments were performed on seeds of *C. arabica* cv ‘Laurina’ in Reunion Island. The experimental plots were planted in 2003 without shade, and were in their second (2006) year of production. Plant spacing was 2 m between rows and 1 m within rows. Among the 107 experimental plots available throughout the island, 16 locations that maximize variation in elevation (270–1032 m asl.) and climatic conditions were selected (Supporting Information Table S1). The survey unit was a compact plot containing about 240 coffee trees. Among the seven developmental stages described previously (Salmona *et al*. 2008), fruits were harvested only at the five last stages that correspond to the development and maturation of the endosperm: ST3 [90–120 days after flowering (DAF)]: aqueous endosperm tissue growing and progressively replacing the perisperm in the locule; ST4 (120–150 DAF): soft milky endosperm; ST5 (150–210 DAF): hard white endosperm while the remaining perisperm resembles a thin green pellicle surrounding the endosperm; ST6 (210–240 DAF): seed from ripening cherry fruits with pericarp turning yellow; ST7 (>240 DAF): mature seed from cherry fruits with red pericarp turning purple. After cross-section, the stage of development of each fruit was determined based on morphological criteria (Salmona *et al*. 2008; Joët *et al*. 2009). The fruit was then immediately frozen in liquid nitrogen in the field and later stored at −80°C. The seed was separated from fruit tissues after freeze-drying only in order to avoid the fast oxidation that occurs in dissected fresh material. For each of the five developmental stages studied, three independent biological samples (pools of *c*. 200 seeds) were collected at intervals of few days on 20 trees randomly selected in each experimental plot.

### Meteorological observations

Reunion Island hosts a dense meteorological network of more than 50 automated stations dedicated to sugarcane agronomy (this service is under the supervision of Meteo France and CIRAD). Climatic conditions (temperature, rainfall, total irradiance and potential evapotranspiration) were estimated for each plot from records at the nearest meteorological stations, and corrected to take the difference in altitude into account. Temperatures were also recorded locally (under the coffee canopy) using portable temperature recorders.

### Real-time RT-PCR

Among the 38 phenylpropanoid gene isoforms whose expression profile has been recently characterized during seed development (Joët *et al*. 2009), 23 gene isoforms were selected on the basis of their maximal expression level during endosperm development and/or maturation (Supporting Information Table S2). The three independent biological samples were pooled. RNA extraction was then performed twice, and real-time RT-PCR was carried out in duplicate for each extract, leading to four replicates per developmental stage for each gene tested. Total RNA extraction and real-time RT-PCR assays were performed as previously described (Salmona *et al*. 2008). Briefly, first-strand cDNA synthesis was performed using the ImProm-II reverse transcription system (Promega, Madison, WI, USA) using 1 *µ*g of total RNA (treated with RNase-free DNase I; Promega) as a template. PCRs were carried out in an optical 96-well plate with an ABI PRISM 7000 sequence detection system (Applied Biosystems, Foster City, CA, USA), using SYBR Green to monitor dsDNA synthesis. Data were analysed using the SDS 2.1 software (Applied Biosystems) to determine cycle threshold (*Ct*) values. PCR efficiency (*E*) was estimated for each gene with LinReg software (Ramakers *et al*. 2003), and taken into account in all subsequent calculations. In order to obtain an accurate internal normalizer, the expression stability of four potential reference genes have been compared (actin, ubiquitin UBQ10, cyclophylin, spermidine synthase and 40S ribosomal protein; Supporting Information Table S2). Using the GeNorm software (Vandesompele *et al*. 2002), *UBQ10* was shown to be the most stable, as evidenced earlier on similar tissues (Salmona *et al*. 2008). However, post hoc analysis also revealed that the mean expression of the 80 genes tested on this biological material was the best normalizer to correct expression data for differences between samples (such as cellular input, RNA quality and reverse transcription efficiency), provided that inter-PCR run effect is negligible (de Kok *et al*. 2005). Normalization was therefore processed by this way, as the inter-PCR run effect was considered negligible, as tested by the use of *UBQ10* in each plate. Relative expression rates of target gene transcripts were then calculated (Supporting Information Table S1) using the following formula: Fold change = (1 + *E*)^−ΔΔ*Ct*^, where Δ*Ct* = *Ct*_target gene_ – *Ct*_reference genes_, and ΔΔ*Ct* = Δ*Ct*_target gene_ – Δ*Ct*_target gene of maximal expression (stage and experimental plot)_. The stage in a particular plot showing maximal expression was therefore normalized to 1. The specificity of the PCR products generated for each set of primers was described previously (Supporting Information Table S2; Joët *et al*. 2009) and was tested by agarose gel electrophoresis, and sequencing of PCR products.

### CGA determination

The samples were analysed in triplicate (from three different extractions) using a completely random experimental design. About 25 mg of freeze-dried powder was extracted with 25 mL of 70% (v/v) aqueous methanol containing 0.5% sodium bisulphite. The samples were shaken for 16 h at 4 °C in darkness on a stirring table at 200 rpm. The extracts were then treated with 0.25 *µ*L of Carrez reagents I and II each (0.25 m potassium ferrocyanide and 1 m zinc acetate, respectively), successively filtered through Whatman Puradisc 25PP 0.45 and 0.2 *µ*m filters (Whatman, Dassel, Germany), and directly used for high-performance liquid chromatography (HPLC). The LC equipment comprised an LC 508 autosampler (Beckman Coulter, Villepinte, France), an LC 126 pump system (Beckman Coulter) and a PDA detector LC 168 (Beckman Coulter) scanning from 200 to 600 nm. CGA was separated on a UP5ODB–25QK (Interchim, Montluçon, France). Solvent A was phosphoric acid 4 mm, and solvent B was methanol. Total flow rate was 1 mL min^−1^, and the column temperature was set at 35 °C. The gradient profile was a linear increase from 5% B to 75% B in 35 min, then a linear increase to 100% B in 5 min, followed by a 5 min isocratic step, and a return to 5% B in 5 min, followed by a 5 min isocratic step to re-equilibrate. CGA isomers were identified by comparing their retention time, chromatograms at 276 and 325 nm and UV spectrum with the previously obtained results (Ky, Noirot & Hamon 1997). They were measured at 325 nm using the 5-CQA calibration curve.

### Statistics

Hierarchical clustering analysis (HCA) and linear regression analyses were carried out using Statistica (Statsoft, Tulsa, OK, USA). The metabolite data set was standardized prior to performing HCA (Ward's pair-wise grouping method, Euclidian distance). Correlations between climatic variables, transcript and metabolite levels were analysed by linear regression using Pearson's correlation coefficient. The different significance thresholds employed (*P* < 0.05, 0.01 or 0.001) are indicated in the Results where necessary. The Pearson correlation coefficients of the standardized expression profiles were also used to draw unweighted co-expression networks among phenylpropanoid genes using an *R* cut-off of 0.8 with a significance threshold of *P* = 0.001.

## RESULTS

### Effects of developmental and environmental factors on the seed CGA metabolism

**Figure 1:**
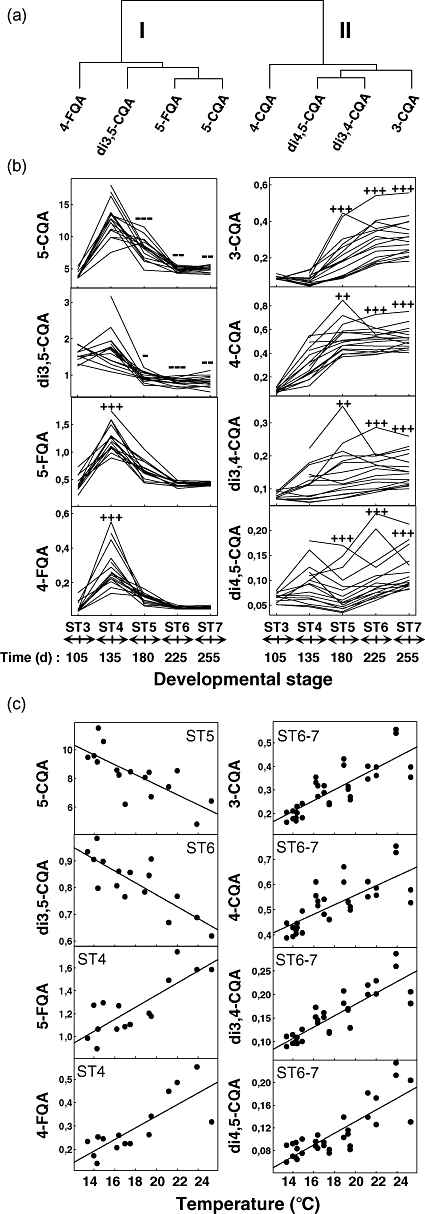
Influence of developmental and environmental factors on chlorogenic acid (CGA) metabolism in the coffee seed. (a) Hierarchical cluster analysis (Euclidian distance, Ward grouping method) of the eight CGA isomers detected according to their profiles in the 16 locations sampled. (b) CGA isomer profiles: the *X*-axis represents seed developmental stages in chronological order, and the *Y*-axis represents the seed content in each CGA expressed as percentage dry mass (DM). For each isomer, the different lines show the accumulation patterns in the 16 experimental plots. Significant positive (+) and negative (−) correlations between air temperature and seed isomer contents are indicated (one symbol, *P* < 0.05; two symbols, *P* < 0.01; three symbols, *P* < 0.001; CQA, caffeoyl quinate; FQA, feruloyl quinate). (c) Correlations (*P* < 0.001) between air temperature and the seed content in the eight isomers. For each isomer, stages showing the highest level of significance (as estimated by *P* and *R* values) were retained to illustrate temperature–CGA correlations.

Eight CGA isomers were identified in developing coffee seeds, independent of the experimental coffee plot sampled (Supporting Information Table S1). For each isomer, the overall accumulation profile was similar among locations, demonstrating that CGA metabolism was controlled by major developmental factors. The eight isomers were clustered in two groups depending on their normalized accumulation pattern (Fig. 1a). Cluster I included the two upstream major isomers, 5-CQA and di3,5-CQA (5 and 1% DM in the mature seed, respectively), and the feruloyl isomers, 4-FQA and 5-FQA. These isomers accumulated very early and within a short period (between stages 3 and 5). This biosynthetic step was followed by a drop in their relative content (as expressed in %DM), mostly because of their subsequent dilution in the growing endosperm (other storage compounds continue to accumulate while CGA synthesis reaches a plateau; Joët *et al*. 2009) and partial remobilization towards lignin biosynthesis. Cluster I compounds showed two types of between-location variations in their profile. Firstly, their transitory maximal content was highly variable. For example, 5-CQA transitory maximal content varied between 7.5 and 18%DM. Secondly, a delay in the biosynthesis peak was detected among locations. For example, in several experimental plots, 5-CQA accumulation stopped at stage 4, while in all the other plots, accumulation remained high until stage 5. Cluster II included the four minor CQA isomers (3-CQA, 4-CQA, di3,4-CQA and di4,5-CQA), which each represented less than 0.8%DM in the mature seed. Unlike cluster I compounds, these CGAs accumulated progressively throughout endosperm development (Fig. 1). Highly significant between-location differences were detected at mid-development (stage 5) for all cluster II isomers. By contrast with minor feruloyl isomers, these differences remained significant until maturity. Although CGA biosynthesis is primarily driven by developmental factors, analysis of clusters I and II profiles clearly revealed that the environment modulates the amplitude of CGA accumulation, as well as the time-window of biosynthetic activities.

### Mean temperature as a proximal cue driving CGA biosynthesis and isomerization

The 16 experimental coffee plots chosen in this study showed very high climatic variations, in particular a very broad temperature gradient, which was highly correlated with altitude (*R* > 0.92, *P* < 10^−4^ for *T*_min_, *T*_mean_ and *T*_max_), which ranged from 150 to 1032 m (Supporting Information Table S1). Relationships between CGA contents and the climatic variables recorded throughout seed development (temperature, rainfall, irradiance and evapotranspiration potential; Supporting Information Table S1) were analysed by linear regression. The mean average air temperature during the last 5 months of seed development (i.e. the period when storage compounds accumulate; Joët *et al*. 2009) showed highly significant correlations with CGA contents at several developmental stages (significant positive and negative correlations are shown in Fig. 1b). By contrast, no significant relationships were found with rainfall or potential evapotranspiration, and only weak correlations were observed with solar irradiance. The dramatic influence of air temperature on the biosynthesis of the different CQA and FQA isomers during fruit growth was again detected at different levels. Firstly, the delay in 5-CQA accumulation observed in several experimental plots was temperature dependent – it occurred in cool locations – as demonstrated by the highly significant negative correlation between 5-CQA content and temperature at stage 5 (Fig. 1c). Secondly, between-location variations in transitory maximal contents of feruloyl isomers were also temperature dependent because at stage 4, the amounts of 5-FQA and 4-FQA were positively correlated with daily temperature (Fig. 1c). Finally, at late stages (ST6-7), the mean air temperature was positively correlated with the amounts of all cluster II isomers (Fig. 1b & 1c), while being negatively correlated with 5-CQA and di3,5-CQA contents (Fig. 1c). This is one of the most important findings of this CGA profiling work: warm climates trigger early accumulation of major upstream CQA isomers, namely 5-CQA and di3,5-CQA, and favour their subsequent isomerization towards minor isomers during late stages.

### Temperature-dependent transcriptional activation of phenylpropanoid genes

**Figure 2:**
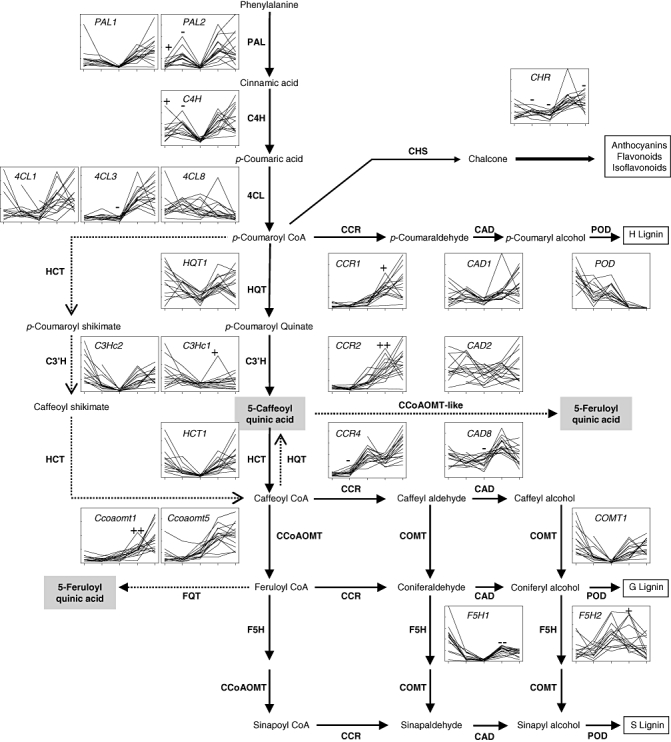
Influence of the environment on the expression profile of genes involved in chlorogenic acid (CGA) and monolignol metabolic pathways. For each gene, the different patterns correspond to the 16 experimental plots, the *X*-axis represents seed developmental stages in chronological order and the *Y*-axis represents the normalized expression value. Significant positive (+) and negative (−) correlations between air temperature and transcript abundance are indicated: one symbol, *P* < 0.05; two symbols, *P* < 0.01. Dashed lines indicate the alternative routes towards 5-caffeoylquinic acid and 5-feruloylquinic acid. C3'H, *p*-coumaroyl CoA 3-hydroxylase; C4H, *trans*-cinnamate 4-hydroxylase; CAD, cinnamyl alcohol dehydrogenase; CCoAOMT, caffeoyl-CoA 3-*O*-methyltransferase; CHS, chalcone synthase; CHR, chalcone reductase; 4CL, 4-coumarate:CoA ligase; CCR, cinnamoyl-CoA reductase; COMT, caffeic acid *O*-methyltransferase; F5H, ferulate 5-hydroxylase; FQT, feruloyl-CoA quinate feruloyl transferase; HCT, hydroxycinnamoyl-CoA:shikimate/quinate hydroxycinnamoyl transferase; HQT, hydroxycinnamoyl-CoA quinate hydroxycinnamoyl transferase; PAL, phenylalanine ammonia lyase; POD, secretory peroxidase.

**Figure 3:**
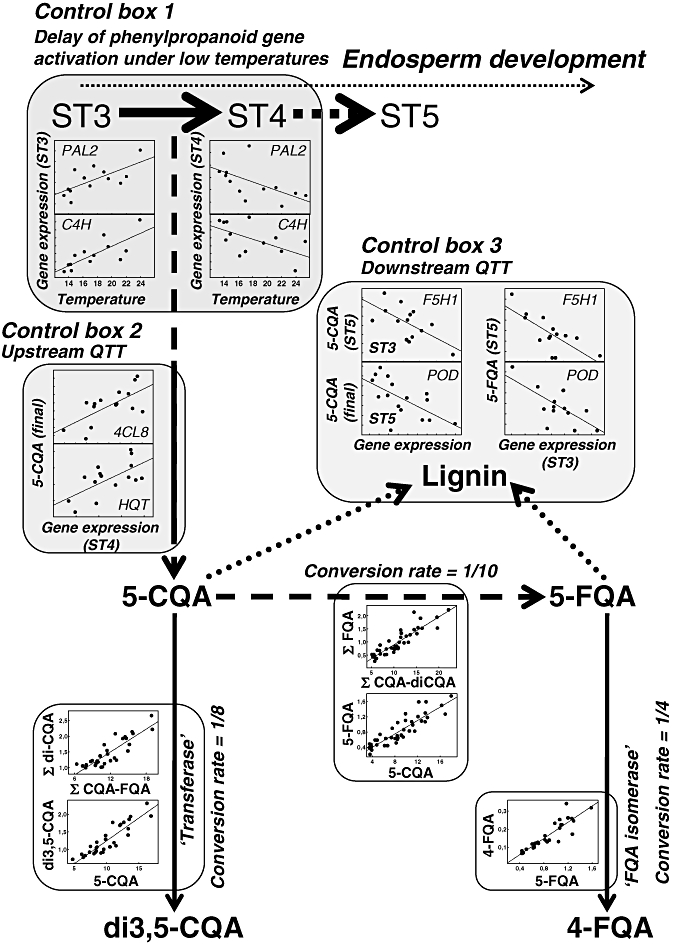
Simplified diagram showing the three ‘control boxes’ of chlorogenic acid (CGA) biosynthesis evidenced in the developing coffee seed: delay in core phenylpropanoid gene activation caused by low temperatures (control box 1), and upstream (2) and downstream (3) Quantitative trait transcripts (QTTs) for 5-CQA and 5-FQA synthesis. Correlations between temperature and transcript abundances, as well as between transcript abundance and metabolite contents, were significant at *P* < 0.05. The isomerization steps characterized by a constant conversion rate over locations and time are also presented. Conversion rates were estimated from the slope of regression lines (*R* > 0.8; *P* < 0.01).

For each of the 23 genes profiled throughout seed development, the global pattern of expression did not depend on the location, again stressing the importance of developmental factors in the transcriptional programmes that interplay during the development of the coffee seed (Fig. 2). However, as for metabolites, we also investigated the influence of environmental factors on transcript accumulation using linear regression. Several highly significant correlations were found between the level of expression of phenylpropanoid genes and mean daily temperature (Fig. 2). For instance, C*CR4* and *CHR* transcript accumulation was negatively correlated with temperature at stage 4. Conversely, in mature stages, the levels of expression of *C3Hc1*, *CCoAOMT1*, *F5H2*, *CCR1* and *CCR2* were positively correlated with temperature. However, the most noticeable result of transcript profiling was the modulation of *PAL2* and *C4H* by temperature in the early stages of endosperm development. Indeed, high temperatures first induced over-accumulation of mRNA encoding these two enzymes of the ‘core’ phenylpropanoid pathway at stage 3 (Fig. 3). The reverse situation (a negative correlation between the level of expression of *PAL2* and *C4H*, and temperature) was then observed at stage 4, indicating a delay in the activation of phenylpropanoid genes under cool climates. This regulation of transcription by temperature represents a first key ‘control box’ of the phenylpropanoid metabolism in the developing coffee seed (Fig. 3). Moreover, it provides a sound explanation for the delay in the accumulation of 5-CQA observed at low temperatures (Fig. 1).

### Transcriptional control of CGA biosynthesis

The variability in the CGA composition of seeds induced by environmental factors constituted a valuable system to test whether the accumulation of the first products of the CQA and FQA pathways (5-CQA and 5-FQA, respectively) is modulated at the transcriptional level. Because these two compounds were synthesized in the early stages of endosperm development, the search for significant quantitative relationships was performed using transcription levels at stages 3–5 only. Highly significant transcript–metabolite correlations (*P* < 0.01) were found with four genes, namely *4CL8*, *HQT*, *F5H1* and *POD* (Fig. 3). Final amounts of 5-CQA were positively correlated with early expression of *HQT* and *4CL8* at stage 4 (*R* = 0.65 and 0.67, respectively; Fig. 3). By contrast, we did not identify any upstream gene whose expression level could explain 5-FQA content. Surprisingly, the expression profile of none of the two *CCoAOMT* genes studied matched with the time-window for FQA accumulation, suggesting that the repertoire of candidate *CCoAOMT* and *OMT* genes should be extended. However, early *F5H1* and *POD* expression was negatively correlated with intermediate amounts of 5-FQA and 5-CQA, showing that transcriptional control of CGA content could also operate at the level of phenylpropanoid genes encoding enzymes involved in their remobilization towards lignin biosynthesis (Fig. 3). Early *F5H1* transcription was negatively correlated with intermediate amounts of 5-FQA and 5-CQA at stage 5 (*R* = −0.70 and −0.64, respectively). Expression of *POD* at stages 3 and 5 was correlated with the intermediate amount of 5-FQA at stage 5 (*R* = −0.67) and the final amount of 5-CQA in mature seed (*R* = −0.63), respectively. These results thus reveal the existence of two other potential ‘control boxes’ of phenylpropanoid metabolism in the developing coffee seed (control boxes 2 and 3 in Fig. 3, respectively).

### Deciphering the isomerization processes through combinatorial analysis

Genes encoding enzymes directly involved in 5-FQA and di3,5-CQA biosynthesis, and those involved in *trans*-esterification of 5-CQA, di3,5-CQA and 5-FQA into minor isomers remain to be identified. However, fine analysis of relationships between the different CGA isomers throughout seed development could provide valuable information about the preferential routes and metabolic regulations that occur during these two processes.

The highly significant correlation found between 5-CQA and 5-FQA during their concomitant biosynthesis suggests that the preferential route for 5-FQA biosynthesis in coffee is likely to be one of the two routes involving 5-CQA as a precursor, therefore excluding a main role for the pathway relying on the synthesis of caffeoyl-CoA through the shikimate ester route. Indeed, if these compounds were the products of two parallel pathways, the chance of identifying a significant relationship between them would be very small.

During their biosynthetic period (stages 3–5), cluster I isomers exhibited highly significant linear correlations (*P* < 0.001) with a very high Pearson's correlation coefficient (*R* > 0.9) (Fig. 3). This result confirms the fact that environmental factors have no impact on the dynamic properties of the isomer conversion processes involved, that is, while the environment significantly influenced the magnitude of 5-CQA biosynthesis, for the downstream isomers concomitantly synthesized from 5-CQA, the estimated rate of conversion at each step was constant (assuming that minor isomers are end products that are not remobilized during seed development, conversion rates could be roughly estimated from ratio between pool sizes, i.e. from the slope of regression lines). Conversion rates were of 1:8 and 1:10 for the conversion of 5-CQA to di3,5-CQA and 5-FQA, respectively (Fig. 3). Further *trans*-esterification of 5-FQA to 4-FQA was characterized by a conversion rate close to 1:4.

**Figure 4:**
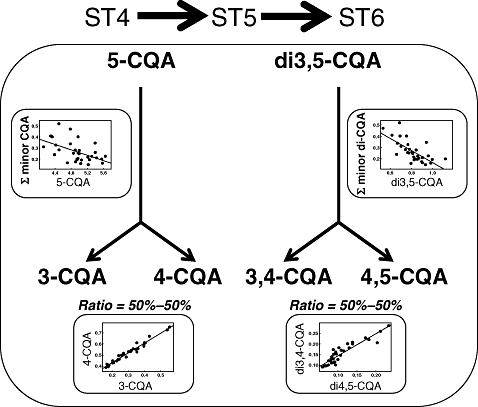
Isomerization to minor CQA: relationships between minor mono- and di-CQA isomers during late stages of development (*R* > 0.8 and *P* < 0.01).

The biosynthesis of minor isomers of mono- and di-CQA was not concomitant with that of their respective precursors, 5-CQA and di3,5-CQA (Fig. 1). Instead, the process of intramolecular isomerization to 3- and 4- positions took place at the end of endosperm development and maturation. Because the pools of 5-CQA and di3,5-CQA were simultaneously partially mobilized for lignin biosynthesis and were not replenished during these late stages, it was not possible to estimate the rates of conversion of 5-CQA and di3,5-CQA to their minor mono- and di-CQA isomers. However, as expected, negative relationships were observed between 5-CQA, and di3,5-CQA and their respective isomers (Fig. 4). In addition, the analysis of the relationships between these minor isomers revealed another characteristic of this metabolism. During the late stages, the slope of the regression line between 3-CQA, and 4-CQA was close to 1 and a *y*-intercept around 0 (Fig. 4). These results indicate that the isomerization process does not present any specificity towards 3- or 4- positions for acylation, leading to the observed biosynthetic ratio of 1:1 for 3-CQA and 4-CQA. A very similar equation was found for the correlation between di3,4-CQA and di4,5-CQA. This balanced ratio clearly indicates that *trans*-esterification of di3,5-CQA could arise from the caffeoyl moiety either on position 3 or 5, leading, respectively, to di3,4-CQA and di4,5-CQA.

### Identifying modules of co-expressed phenylpropanoid genes through quantitative co-expression analysis

**Figure 5:**
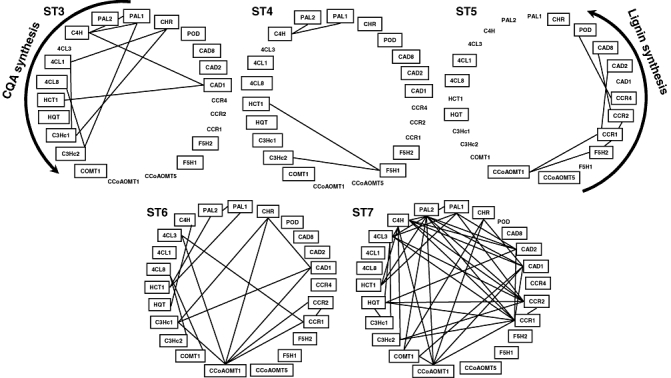
Network of positively co-expressed phenylpropanoid genes during seed development. Modules of co-expression were designed using a *P* value cut-off of 0.001 (corresponding to a Pearson's correlation coefficient >0.80). At each developmental stage, genes actively transcribed (expression value >0.1) are boxed. Significant correlations between the transcript abundances are shown by a straight line.

The present transcript profiling work showed that several sets of genes shared a similar pattern (Fig. 2), suggesting their temporal co-expression. Groups of co-expressed genes are classically identified by HCA, *K*_means_ and other clustering methods (e.g. Gachon *et al*. 2005; Mutwil *et al*. 2010). However, clustering cannot test whether the co-expression of two genes is also quantitative or not. Thanks to the quantitative variation in transcription level induced by the environment, the present data constitute a valuable system to test the existence of networks of phenylpropanoid genes that are quantitatively co-expressed or co-repressed. This was done through extensive pair-wise correlation analysis using stringent Pearson correlation significance thresholds (*P* < 0.001; *R* > 0.8). For each developmental stage, a synthetic representation of the network of co-expressed genes obtained is given in Fig. 5. A close examination of these networks revealed the existence of three independent modules of quantitatively and temporally co-expressed genes throughout seed development. The first module was activated very early (ST3) and comprised genes encoding upstream phenylpropanoid biosynthetic enzymes: *PAL1*, *PAL2*, *C4H*, *4CL1*, *4CL8*, *C3Hc1*, *C3Hc2* and *HCT1* (Fig. 5). This result is consistent with our observation that the biosynthesis peak of the major CGA occurred at stages 3 and 4 (Fig. 1). The second module included many genes potentially involved in CGA mobilization and lignin biosynthesis, namely *CCoAOMT1*, *F5H2*, *CCR1*, *CCR2*, *CCR4*, *CAD2*, *CAD8* and *POD*, and was triggered at stage 5. Again, the activation of this catabolic module at mid-development is coherent with the partial remobilization of CGA at this stage (Fig. 1), which was also previously shown to correspond to the period of cell wall thickening (Joët *et al*. 2009). Finally, in maturing seeds, almost all phenylpropanoid genes under study were quantitatively co-expressed, leading to a very dense network at stage 7 (Fig. 5). In this network, the two key genes of the ‘core’ phenylpropanoid pathway, namely *PAL2* and *C4H*, presented the highest number of significant connections. The quantitative modulation of this third network across environments was apparently not connected with the anabolism or remobilization of CGA during seed maturation.

## DISCUSSION

### Modulation of phenylpropanoid metabolism by temperature

Long-term field experiments to test the effects of temperature are usually interpreted with caution because several uncontrolled environmental factors may interplay during changes in the season and drive complex metabolic regulations. However, tropical climates are fortunately characterized by very slight variations in seasonal temperature and light. Elevation is thus the main factor modulating temperature in tropical areas. Consequently, in our experimental design, differences in temperature between locations were very well conserved throughout the fruit development period. Without a doubt, the stability of inter-plot differences in temperature over time made our design a powerful tool to decipher the effects of temperature on CGA metabolism. Indeed, although seed CGA metabolism is driven by major developmental factors (Joët *et al*. 2009), environmental temperature was also shown to have a direct impact on the amplitude of CGA accumulation, as well as the time-window of biosynthetic activities through subtle transcriptional, and possibly enzymatic, regulations. Among these regulations, *PAL2* and *C4H* mRNA accumulation was shown to be temperature dependent in early developmental stages, and associated with a delay in phenylpropanoid synthesis under cool climates. At the stage of maximal CGA biosynthetic activity (ST4), *PAL2* and *C4H* were up-regulated by low temperatures. Similarly, in *Arabidopsis*, the modulation of tissue phenolic composition by temperature is primarily controlled at the transcriptional level (Vogel *et al*. 2005; Usadel *et al*. 2008). Moreover, like in coffee, *PAL* and other phenylpropanoid genes are also rapidly up-regulated in response to low temperatures in *Arabidopsis* (Olsen *et al*. 2008).

### Identification of QTTs for CGA accumulation

The high concordance between transcript and metabolite profiles revealed in a previous work (Joët *et al*. 2009) was the first indication that CGA metabolism may be mainly controlled at the transcriptional level in the developing coffee seed. In the present work, we showed that low temperatures delayed both transcriptional activation of *PAL* and *C4H*, and accumulation of 5-CQA. This result provides an additional clue that CGA metabolism may be controlled at the transcriptional level in the developing coffee seed. To further substantiate this hypothesis, we searched for correlations between transcript abundance and seed contents in the two major CGA, namely 5-CQA and 5-FQA. Our data clearly evidenced quantitative relationships between *HQT*, *4CL8*, *F5H1* and *POD* transcript abundance in the developing coffee endosperm and final 5-CQA amounts, revealing that the respective encoded enzymes may play a significant role in the control of CGA biosynthesis fluxes in coffee. Passador-Gurgel *et al*. (2007) recently proposed to refer to such transcript–phenotypic trait associations as QTTs.

In other plants, enzymes of the ‘core phenylpropanoid pathway’ have often been described as the best candidates for flux control in CGA metabolism (Bate *et al*. 1994; Dixon & Paiva 1995; Blount *et al*. 2000). As the bridge between primary metabolism and phenolic compound biosynthesis, PAL, in particular, was often proposed for pathway regulation and was shown to be the rate-determining step in the synthesis of CGA in tobacco leaves, with a flux control coefficient close to unity (Bate *et al*. 1994; Howles *et al*. 1996). In coffee seeds, our data evidenced that *HQT* and *4CL8* are QTT for 5-CQA biosynthesis, therefore excluding an exclusive role for PAL. In tomato fruits, it was also shown that PAL was not the only enzyme regulating CGA accumulation (Niggeweg *et al*. 2004). Moreover, our data indicate that *F5H1* and *POD* are QTTs for 5-CQA remobilization and remobilization towards lignin. This result clearly demonstrates that when the storage compound is also an intermediary product, downstream regulation warrants as much attention as that occurring upstream to get a reliable overview of the whole metabolic process. Search for QTT was also shown to be of great help in identifying rate-controlling steps in other particular pathways. For instance, transcript levels of seven genes of the carotenoid pathway were found to positively or negatively correlate with endosperm carotenoid content in genetically diverse maize germplasm (Vallabhaneni & Wurtzel 2009). However, to our knowledge, this is the first time that variations induced by the environment in a single genotype have been successfully used to identify QTT.

Overall, the above results indicate that for some major seed storage compounds, transcriptional control alone may explain a large proportion of variations in phenotypic traits. However, we assume that post-transcriptional regulations could play a significant role in one of the final stages of their synthesis (i.e. *trans*-esterification of 5-CQA and di3,5-CQA into minor CQA and di-CQA isomers). We clearly showed that warm climates favoured these *trans*-esterification processes. Interestingly, a comparable temperature-dependent CGA isomerization process was recently reported in sweet potato tubers when they were gradually heated (Takenaka *et al*. 2006). These reactions were shown to be enzymatically driven. The short time-course of the experiment (<20 min) excluded significant transcriptional control. It is therefore plausible that *trans*-esterification enzyme activities are also influenced by temperature in the developing coffee seed. However, one may also hypothesize that these chemical modifications arise without enzymatic control as spontaneous isomerization of 5-CQA has been evidenced upon heating (Hanson 1965).

Finally, the highly significant correlation found between intermediate amounts of 5-FQA and 5-CQA suggests that feruloyl quinate directly derives from 5-CQA and not from the parallel shikimate ester route. The molecular characterization of FQA biosynthetic enzymes, *O*-methyl transferase and/or acyl-transferase, will be an important step to validate this hypothesis. With this in view, our observation that 5-FQA accumulation was also driven in a temperature-dependent way opens a new avenue for the molecular identification through co-expression analysis of the genes involved in the two potential FQA biosynthetic routes that use 5-CQA as precursor. Co-expression analysis by regression of microarray data has already been successfully used for the identification of cellulose synthase subunits (Persson *et al*. 2005), new flavonoid biosynthetic genes (Yonekura-Sakakibara *et al*. 2008) and drought-related genes (Lee *et al*. 2009). In this respect, the current building of 15k oligonucleotide microarrays dedicated to coffee genomics (Privat, Bertrand & Lashermes 2008) and coffee genome sequencing opens new perspectives.

### Stage-specific modular organization of the phenylpropanoid gene expression network

In recent studies, search for modules of co-expressed genes was often performed at the quantitative level through regression analysis (e.g. Wei *et al*. 2006; Yonekura-Sakakibara *et al*. 2008). Indeed, in addition to the information provided by clustering analysis, the detection of significant correlations between transcript abundance of genes of a given module is a further step in the demonstration that these genes are co-regulated (Saito, Hirai & Yonekura-Sakakibara 2008; Usadel *et al*. 2009). This approach was mostly used to analyse compilations of microarray data that encompassed a wide range of experimental conditions (Wei *et al*. 2006; Srinivasasainagendra *et al*. 2008; Lee *et al*. 2009). Here, we used it for the description of the fine transcriptional regulation of phenylpropanoid genes by the environment, and we evidenced three modules of quantitatively co-expressed genes, temporally non-overlapping. This restricted number of modules is in agreement with the modular organization of *Arabidopsis* flavonoid metabolism (Gachon *et al*. 2005) and lignin biosynthesis pathway (Hertzberg *et al*. 2001), as uncovered by HCA. The present work also highlights the facts that co-expression modules can be restricted to specific developmental stages of a given tissue and that they could be easily revealed through the survey of a restricted gene repertoire using real-time RT-PCR.

The gene composition of the first two modules we identified – CGA biosynthesis and remobilization, respectively, – corresponded precisely with the composition of the two clusters of co-expressed genes previously identified by HCA of expression profiles (Joët *et al*. 2009). However, the present data also revealed the existence of a third module of quantitatively co-expressed genes during the final developmental stage whose role remains to be elucidated. This module included most of the phenylpropanoid genes studied. It could either be involved in the synthesis of phenylpropanoid derivatives other than CGA during maturation, such as volatile phenylpropenes, synthesized in ripening fruits and seeds (Koeduka *et al*. 2009; Tikunov *et al*. 2010), or represent a set of stored mRNA that may be translated later during seed germination. Indeed, mRNA stored in the quiescent seed was proved to play a crucial role during seed germination (Rajjou *et al*. 2004). Genome-wide investigation of mRNA storage in quiescent *A. thaliana* seeds revealed that more than 12 000 mRNA species are stored, including all ontological categories and, in particular, most of the phenylpropanoid genes that are orthologous to the coffee genes studied here (Nakabayashi *et al*. 2005).

As mentioned above, the identification through regression analysis of modules of co-expressed phenylpropanoid genes in the developing coffee seed strongly suggests that the genes in each module are co-regulated. The next step of the present work is undoubtedly to search for transcription factors regulating phenylpropanoid gene expression during coffee seed development. In maize, the branches of the phenylpropanoid pathway leading to flavonols and CGA are co-regulated by the p1 locus, which encodes an myb-like transcriptional activator (Grotewold *et al*. 1994; Bushman *et al*. 2002; Szalma *et al*. 2005). Similarly, in tomato fruits, a transcription factor orthologous to the *A. thaliana* flavonol-specific transcriptional activator AtMYB12 probably co-activates both CGA and flavonol biosynthetic pathways, because the ectopic expression of AtMYB12 led to the accumulation 20-fold higher levels of both products (Luo *et al*. 2008). Interestingly, in our experiments, chalcone reductase, which belongs to the flavonoid branch, joined the phenylpropanoid biosynthesis module, suggesting that flavonoid and CGA biosynthesis could be also co-regulated during coffee seed development. It is tempting to speculate that an MYB12-like transcriptional activator is also present in coffee. Provided that microarray data sets are available, co-expression analysis, either by HCA or regression, has proved to be a powerful tool for identifying regulatory elements of a particular metabolic pathway (Hirai *et al*. 2007). Again, genome sequencing and microarray building initiatives in coffee should allow this task to be achieved in the near future.

## SUPPORTING INFORMATION

**Table S1.** Chlorogenic acid (CGA) composition and phenylpropanoid gene expression in developing coffee seeds grown in various environments. Climatic data for the 16 experimental plots are also listed.

**Table S2.** List of specific primers used for real-time RT-PCR.
